# Sex, age, and ethnic dependency of lipoprotein variants as the risk factors of ischemic heart disease: a detailed study on the different age-classes and genders in Tehran Cardiometabolic Genetic Study (TCGS)

**DOI:** 10.1186/s13293-022-00413-7

**Published:** 2022-01-28

**Authors:** Hossein Lanjanian, Leila Najd Hassan Bonab, Mahdi Akbarzadeh, Maryam Moazzam-Jazi, Asiyeh Sadat Zahedi, Sajedeh Masjoudi, Maryam S. Daneshpour

**Affiliations:** Cellular and Molecular Endocrine Research Center, Research Institute for Endocrine Sciences, Shahid Beheshti University of Medical Sciences, No 24, Parvaneh St, Yemen St, Chamran Exp, PO Box 1985717413, Tehran, Iran

**Keywords:** TCGS, Lipoprotein (a), Lp(a), *LPA* locus, Single nucleotide polymorphism, Myocardial infarction (MI), Age-of-onset, Sex, Age

## Abstract

Biological processes involving environmental and genetic factors drive the interplay between age- and sex-regulating lipid profile. The relation between variations in the *LPA* gene with increasing the risk of coronary heart disease is dependent on population differences, sex, and age. The present study tried to do a gene candidate association analysis in people with myocardial infarction (MI) in a 22 year cohort family-based longitudinal cohort study, Tehran Cardiometabolic Genetic Study (TCGS). After adjusting *p *value by the FDR method, only the association of rs6415084 with the MI probability and the age-of-CHD-onset was significant in males in their middle age (*p* < 0.005). Surprisingly, a lack of association was observed for the rest of the markers (16 SNPs). These results revealed the moderator effects of age and sex on the association between the genetic variants (SNPs) of *LPA* and heart disease risk. Our observations may provide new insights into the biology that underlies lipid profile with age or the sexual dimorphism of Lp(a) metabolism. Finally, Lp(a) appears to be an independent risk factor; however, the role of sex and ethnicity is important.

**Supplementary Information:**

The online version contains supplementary material available at 10.1186/s13293-022-00413-7.

## Introduction

Elevated lipoprotein (a) is associated with incidence and severity increasing of cardiovascular diseases [1, 2]. In the Chinese Han population, the association of five SNPs (rs1367211, rs3127596, rs9347438, rs6415085, and rs9364559) in the *LPA* gene with the development of coronary artery disease (CAD) were evaluated and also rs10455872 in predicting the risk of CHD events in statin therapy plays a significant role [3, 4]. Among these variants, rs6415085 was also associated with the increased *LPA* expression level and coronary artery disease (CAD) [5]. Rs3798220 and rs10455872 were associated with Lp(a) concentration and CAD prevalence [6, 7]. In assessing the prevalence of rs3798220 and rs10455872 polymorphisms of *LPA* gene in the subpopulation of patients with symptomatic and asymptomatic carotid stenosis, only a significant association was observed between rs3798220 polymorphism and carotid artery stenosis incidence. Contrastingly, no association was detected for rs3798220 and rs10455872 and atherogenic stroke [8]. A study in a large sample of Brazilian patients confirmed the association of rs10455872 with CAD development, while it showed a lack of association of the rs3798220 with this disease [9]. Heinz Nixdorf Recall’s study provided evidence for the association of *LPA* rs10455872 with higher Lp(a) and Coronary artery calcification (CAC), a well-proven marker for coronary artery disease and a risk factor for cardiovascular events. [10].

However, the investigation of tertiles Lipoprotein (a) concentration, rs10455872, and rs3798220 with all-cause mortality and cardiovascular mortality with the severity of disease in a large-scale study showed that lipoprotein (a) concentrations and the genetic variants have no associations with mortality in patients with prevalent coronary heart disease. The results showed that these variables are not useful risk factors to predict progression to death after coronary heart disease is established [11].

The Lv et al. study did not display any significant evidence of four SNPs' associations (rs2048327, rs3127599, rs7767084, and rs10755578) *SLC22A3-LPAL2-LPA* gene cluster with CAD in a large Chinese Han sample [12].

Lack of association between lipoprotein (a) genetic variants (rs6415084 and rs3798220) and subsequent cardiovascular events in Chinese Han patients with coronary artery disease after the percutaneous coronary intervention has also been reported [13].

To resolve ambiguity and investigate the between-population differences in Lp(a) levels, we refer to the Dumitrescu et al. study, which genotyped 19 LP(A) tag SNPs in 7159 participants from the Third National Health and Nutrition Examination Survey (NHANES III). Notably, there were more significant associations between Lp(a) and LP(A) SNPs in non-Hispanic blacks than non-Hispanic whites and Mexican Americans. Moreover, nearly, half of these associations were exclusive to non-Hispanic blacks [14]. *LPA* SNPs' prevalence and association with the size of apolipoprotein(a) isoforms, Lp(a), and OxPL-apoB levels are highly variable and ethnicity-specific. *LPA* SNP rs3798220 was most prevalent in Hispanics (42.38%), rs10455872 in whites (14.27%), and rs9457951 in blacks (32.92%). The correlation of each of these SNPs with the major apolipoprotein(a) isoform size was highly variable and in different directions among ethnic groups [15].

The current case–control study examines the association between 17 observed *LPA* polymorphisms with myocardial infarction (MI) risk across the Tehran Cardiometabolic Genetic Study (TCGS) participants. Our analysis considered the effect of age and sex in the process of evaluating this relationship.

## Material and methods

In the present case–control study, 783 unrelated individuals with MII were selected and compared with the same number of controls. These individuals were assigned from the Tehran Cardiometabolic Genetic Study (TCGS), which is a large-scale family-based longitudinal cohort study [16] that is a subpopulation of the Tehran Lipid and Glucose Study (TLGS) [17]. TLGS is and community-based cohort study on fifteen thousand people launched in 1999 in the 13th district of Tehran. The first survey of the TLGS was initiated from 1999 to 2001 on 15,005 individuals aged 3 years, and subjects were genotyped and followed up to identify recently developed diseases every 3 years. The research council of the Endocrine Research Center of the Shahid Beheshti University of Medical Sciences approved the study.

At each survey of TLGS, participants signed a consent form. A standardized questionnaire collected information for age, sex, and history of using medication for diabetes, hypertension, and lipid disorders. Anthropometric measurements, including weight (kg), height (cm), and waist circumference (cm) recorded using standard protocols. Body mass index (BMI) is calculated as weight in kilograms divided by height in square meters. Systolic blood pressure (SBP), diastolic blood pressure (DBP) were measured as described previously [18]. A blood sample draws after 12–14 h overnight fasting. Samples were centrifuged within 30–45 min of collection, and the sera were used for biochemical measurements. Serum glucose, TC, TG, and HDL-C were measured using commercial kits using the enzymatic colorimetric method (Pars Azmoon, Tehran, Iran). Coefficients of variation (CV) for total cholesterol, HDL_C, and triglyceride measurements were below 5%. LDL_C concentrations were calculated using a modified Friedewald equation. Fasting plasma glucose (FPG), triglycerides (TG), total cholesterol (TC), and high-density lipoprotein cholesterol (HDL_C) levels were measured by Pars AzmunCo (Iran); also, coefficients of variation (CV) for total cholesterol, HDL_C and triglyceride measurements were below 5%. Non-HDL_C calculate by subtracting HDL_C from TC. LDL_C concentrations were computed using a modified Friedewald’s equation [19].

In this study, to evaluate the associations between these factors and genetic markers, the obtained values in the last measurement phase before the MI of each case (pre-MI phase) were considered.

### Genetic analysis

Genomic samples were extracted from the buffy coat using the standard Proteinase K, the salting-out method. DNA samples were genotyped with HumanOmniExpress-24-v1-0 bead chips (containing 649,932 SNP loci with an average mean distance of 4 kb) at the deCODE genetics company (Iceland) according to the manufacturer’s specifications (Illumina Inc., San Diego, CA, USA). The PLINK program (V 1.07) [20] and R statistic (V 3.2) performed quality control procedures. The genotype information for 17 selected markers in *LPA* gene (rs7449650, rs11751605, rs7761293, rs6415084, rs9365171, rs7770628, rs6926458, rs6930542, rs13202636, rs7761377, rs10945682, rs7756317, rs1321196, rs1367211, rs9346833, rs783149, and rs1084651) was extracted for the studied population after performing quality control procedures.

The participants diagnosed with coronary heart disease until 2017 were selected as the case group (CHD) for the current study during the follow-up time [21]. We selected an unrelated participant of the same sex and age for each case by in-house python programming to control selection (non-CHD). The control participants also had no history of cardiovascular disease, diabetes mellitus, or metabolic syndrome. All of the controls and cases are unrelated, and they were not belong to the same big family since nearly all of these participants live in a specific region of the Tehran capital city, so they experienced the same environmental and pollution conditions. ‬‬‬‬‬‬‬‬‬‬‬‬‬‬‬‬‬‬‬‬‬‬‬‬‬‬‬‬‬‬‬‬‬‬‬‬‬‬‬‬‬‬‬‬‬‬‬‬‬‬‬‬‬‬‬‬‬‬‬‬‬‬‬‬‬‬‬‬‬‬‬‬‬‬‬‬‬‬‬‬‬‬‬‬‬‬‬‬‬‬‬‬‬‬‬‬‬‬‬‬‬‬‬‬‬‬‬‬‬‬‬‬‬‬‬‬‬‬‬‬‬‬‬‬‬‬‬‬‬‬‬‬‬‬‬‬‬‬‬‬‬‬‬‬‬‬‬‬‬‬‬‬‬‬‬‬‬‬‬‬‬‬‬‬‬‬‬‬‬‬‬‬‬‬‬‬‬‬‬‬‬‬‬‬‬‬‬‬‬‬‬‬‬‬‬‬‬‬‬‬‬‬‬‬‬‬‬‬‬‬‬‬‬‬‬‬‬‬‬‬‬‬‬‬‬‬‬‬‬‬‬‬‬‬‬‬‬‬‬‬‬‬‬‬‬‬‬‬‬‬‬‬‬‬‬‬‬‬‬‬‬‬‬‬‬‬‬‬‬‬‬‬‬‬‬‬‬‬‬‬‬‬‬‬‬‬‬‬‬‬‬‬‬‬‬‬‬‬‬‬‬‬‬‬‬‬‬‬‬‬‬‬‬‬‬‬‬‬‬‬‬‬‬‬‬‬‬‬‬‬‬‬‬‬‬‬‬‬‬‬‬‬‬‬‬‬‬‬‬‬‬‬‬‬‬‬‬‬‬‬‬‬‬‬‬‬‬‬‬‬‬‬‬‬‬‬‬‬‬‬‬‬‬‬‬‬‬‬‬‬‬‬‬‬‬‬‬‬‬‬‬‬‬‬‬‬‬‬‬‬‬‬‬‬‬‬‬‬‬‬‬‬‬‬‬‬‬‬‬‬‬‬‬‬‬‬‬‬‬‬‬‬‬‬‬‬‬‬‬‬‬‬‬‬‬‬‬‬‬‬‬‬‬‬‬‬‬‬‬‬‬‬‬‬‬‬‬‬‬‬‬‬‬‬‬‬‬‬‬‬‬‬‬‬‬‬‬‬‬‬‬‬‬‬‬‬‬‬‬‬‬‬‬‬‬‬‬‬‬‬‬‬‬‬‬‬‬‬‬‬‬‬‬‬‬‬‬‬‬‬‬‬‬‬‬‬‬‬‬‬‬‬‬‬‬‬‬‬‬‬‬‬‬‬‬‬‬‬‬‬‬‬‬‬‬‬‬‬‬‬‬‬‬‬‬‬‬‬‬‬‬‬‬‬‬‬‬‬‬‬‬‬‬‬‬‬‬‬‬‬‬‬‬‬‬‬‬‬‬‬‬‬‬‬‬‬‬‬‬‬‬‬‬‬‬‬‬‬‬‬‬‬‬‬‬‬‬‬‬‬‬‬‬‬‬‬‬‬‬‬‬‬‬‬‬‬‬‬‬‬‬‬‬‬‬‬‬‬‬‬‬‬‬‬‬‬‬‬‬‬‬‬‬‬‬‬‬‬‬‬‬‬‬‬‬‬‬‬‬‬‬‬‬‬‬‬‬‬‬‬‬‬‬‬‬‬‬‬‬‬‬‬‬‬‬‬‬‬‬‬‬‬‬‬‬‬‬‬‬‬‬‬‬‬‬‬‬‬‬‬‬‬‬‬‬‬‬‬‬‬‬‬‬‬‬‬‬‬‬‬‬‬‬‬‬‬‬‬‬‬‬‬‬‬‬‬‬‬‬‬‬‬‬‬‬‬‬‬‬‬‬‬‬‬‬‬‬‬‬‬‬‬‬‬‬‬‬‬‬‬‬‬‬‬‬‬‬‬‬‬‬‬‬‬‬‬‬‬‬‬‬‬‬‬‬‬‬‬‬‬‬‬‬‬‬‬‬‬‬‬‬‬‬‬‬‬‬‬‬‬‬‬‬‬‬‬‬‬‬‬‬‬‬‬‬‬‬‬‬‬‬‬‬‬‬‬‬‬‬‬‬‬‬‬‬‬‬‬‬‬‬‬‬‬‬‬‬‬‬‬‬‬‬‬‬‬‬‬‬‬‬‬‬‬‬‬‬‬‬‬‬‬‬‬‬‬‬‬‬‬‬‬‬‬‬‬‬‬‬‬‬‬‬‬‬‬‬‬‬‬‬‬‬‬‬‬‬‬‬‬‬‬‬‬‬‬‬‬‬‬‬‬‬‬‬‬‬‬‬‬‬‬‬‬‬‬‬‬‬‬‬‬‬‬‬‬‬‬‬‬‬‬‬‬‬‬‬‬‬‬‬‬‬‬‬‬‬‬‬‬‬‬‬‬‬‬‬‬‬‬‬‬‬‬‬‬‬‬‬‬‬‬‬‬‬‬‬‬‬‬‬‬‬‬‬‬‬‬‬‬‬‬‬‬‬‬‬‬‬‬‬‬‬‬‬‬‬‬‬‬‬‬‬‬‬‬‬‬‬‬‬‬‬‬‬‬‬‬‬‬‬‬‬‬‬‬‬‬‬‬‬‬‬‬‬‬‬‬‬‬‬‬‬‬‬‬‬‬‬‬‬‬‬‬‬‬‬‬‬‬‬‬‬‬‬‬‬‬‬‬‬‬‬‬‬‬‬‬‬‬‬‬‬‬‬‬‬‬‬‬‬‬‬‬‬‬‬‬‬‬‬‬‬‬‬‬‬‬‬‬‬‬‬‬‬‬‬‬‬‬‬‬‬‬‬‬‬‬‬‬‬‬‬‬‬‬‬‬‬‬‬‬‬‬‬‬‬‬‬‬‬‬‬‬‬‬‬‬‬‬‬‬‬‬‬‬‬‬‬‬‬‬‬‬‬‬‬‬‬‬‬‬‬‬‬‬‬‬‬‬‬‬‬‬‬‬‬‬‬‬‬‬‬‬‬‬‬‬‬‬‬‬‬‬‬‬‬‬‬‬‬‬‬‬‬‬‬‬‬‬‬‬‬‬‬‬‬‬‬‬‬‬‬‬‬‬‬‬‬‬‬‬‬‬‬‬‬‬‬‬‬‬‬‬‬‬‬‬‬‬‬‬‬‬‬‬‬‬‬‬‬‬‬‬‬‬‬‬‬‬‬‬‬‬‬‬‬‬‬‬‬‬‬‬‬‬‬‬‬‬‬‬‬‬‬‬‬‬‬‬‬‬‬‬‬‬‬‬‬‬‬‬‬‬‬‬‬‬‬‬‬‬‬‬‬‬‬‬‬‬‬‬‬‬‬‬‬‬‬‬‬‬‬‬‬‬‬‬‬‬‬‬‬‬‬‬‬‬‬‬‬‬‬‬‬‬‬‬‬‬‬‬‬‬‬‬‬‬‬‬‬‬‬‬‬‬‬‬‬‬‬‬‬‬‬‬‬‬‬‬‬‬‬‬‬‬‬‬‬‬‬‬‬‬‬‬‬‬‬‬‬‬‬‬‬‬‬‬‬‬‬‬‬‬‬‬‬‬‬‬‬‬‬‬‬‬‬‬‬‬‬‬‬‬‬‬‬‬‬‬‬‬‬‬‬‬‬‬‬‬‬‬‬‬‬‬‬‬‬‬‬‬‬‬‬‬‬‬‬‬‬‬‬‬‬‬‬‬‬‬‬‬‬‬‬‬‬‬‬‬‬‬‬‬‬‬‬‬‬‬‬‬‬‬‬‬‬‬‬‬‬‬‬‬‬‬‬‬‬‬‬‬‬‬‬‬‬‬‬‬‬‬‬‬‬‬‬‬‬‬‬‬‬‬‬‬‬‬‬‬‬‬‬‬‬‬‬‬‬‬‬‬‬‬‬‬‬‬‬‬‬‬‬‬‬‬‬‬‬‬‬‬‬‬‬‬‬‬‬‬‬‬‬‬‬‬‬‬‬‬‬‬‬‬‬‬‬‬‬‬‬‬‬‬‬‬‬‬‬‬‬‬‬‬‬‬‬‬‬‬‬‬‬‬‬‬‬‬‬‬‬‬‬‬‬‬‬‬‬‬‬‬‬‬‬‬‬‬‬‬‬‬‬‬‬‬‬‬‬‬‬‬‬‬‬‬‬‬‬‬‬‬‬‬‬‬‬‬‬‬‬‬‬‬‬‬‬‬‬‬‬‬‬‬‬‬‬‬‬‬‬‬‬‬‬‬‬‬‬‬‬‬‬‬‬‬‬‬‬‬‬‬‬‬‬‬‬‬‬‬‬‬‬‬‬‬‬‬‬‬‬‬‬‬‬‬‬‬‬‬‬‬‬‬‬‬‬‬‬‬‬‬‬‬‬‬‬‬‬‬‬‬‬‬‬‬‬‬‬‬‬‬‬‬‬‬‬‬‬‬‬‬‬‬‬‬‬‬‬‬‬‬‬‬‬‬‬‬‬‬‬‬‬‬‬‬‬‬‬‬‬‬‬‬‬‬‬‬‬‬‬‬‬‬‬‬‬‬‬‬‬‬‬‬‬‬‬‬‬‬‬‬‬‬‬‬‬‬‬‬‬‬‬‬‬‬‬‬‬‬‬‬‬‬‬‬‬‬‬‬‬‬‬‬‬‬‬‬‬‬‬‬‬‬‬‬‬‬‬‬‬‬‬‬‬‬‬‬‬‬‬‬‬‬‬‬‬‬‬‬‬‬‬‬‬‬‬‬‬‬‬‬‬‬‬‬‬‬‬‬‬‬‬‬‬‬‬‬‬‬‬‬‬‬‬‬‬‬‬‬‬‬‬‬‬‬‬‬‬‬‬‬‬‬‬‬‬‬‬‬‬‬‬‬‬‬‬‬‬‬‬‬‬‬‬‬‬‬‬‬‬‬‬‬‬‬‬‬‬‬‬‬‬‬‬‬‬‬‬‬‬‬‬‬‬‬‬‬‬‬‬‬‬‬‬‬‬‬‬‬‬‬‬‬‬‬‬‬‬‬‬‬‬‬‬‬‬‬‬‬‬‬‬‬‬‬‬‬‬‬‬‬‬‬‬‬‬‬‬‬‬‬‬‬‬‬‬‬‬‬‬‬‬‬‬‬‬‬‬‬‬‬‬‬‬‬‬‬‬‬‬‬‬‬‬‬‬‬‬‬‬‬‬‬‬‬‬‬‬‬‬‬‬‬‬‬‬‬‬‬‬‬‬‬‬‬‬‬‬‬‬‬‬‬‬‬‬‬‬‬‬‬‬‬‬‬‬‬‬‬‬‬‬‬‬‬‬‬‬‬‬‬‬‬‬‬‬‬‬‬‬‬‬‬‬‬‬‬‬‬‬‬‬‬‬‬‬‬‬‬‬‬‬‬‬‬‬‬‬‬‬‬‬‬‬‬‬‬‬‬‬‬‬‬‬‬‬‬‬‬‬‬‬‬‬‬‬‬‬‬‬‬‬‬‬‬‬‬‬‬‬‬‬‬‬‬‬‬‬‬‬‬‬‬‬‬‬‬‬‬‬‬‬‬‬‬‬‬‬‬‬‬‬‬‬‬‬‬‬‬‬‬‬‬‬‬‬‬‬‬‬‬‬‬‬‬‬‬‬‬‬‬‬‬‬‬‬‬‬‬‬‬‬‬‬‬‬‬‬‬‬‬‬‬‬‬‬‬‬‬‬‬‬‬‬‬‬‬‬‬‬‬‬‬‬‬‬‬‬‬‬‬‬‬‬‬‬‬‬‬‬‬‬‬‬‬‬‬‬‬‬‬‬‬‬‬‬‬‬‬‬‬‬‬‬‬‬‬‬‬‬‬‬‬‬‬‬‬‬‬‬‬‬‬‬‬‬‬‬‬‬‬‬‬‬‬‬‬‬‬‬‬‬‬‬‬‬‬‬‬‬‬‬‬‬‬‬‬‬‬‬‬‬‬‬‬‬‬‬‬‬‬‬‬‬‬‬‬‬‬‬‬‬‬‬‬‬‬‬‬‬‬‬‬‬‬‬‬‬‬‬‬‬‬‬‬‬‬‬‬‬‬‬‬‬‬‬‬‬‬‬‬‬‬‬‬‬‬‬‬‬‬‬‬‬‬‬‬‬‬‬‬‬‬‬‬‬‬‬‬‬‬‬‬‬‬‬‬‬‬‬‬‬‬‬‬‬‬‬‬‬‬‬‬‬‬‬‬‬‬‬‬‬‬‬‬‬‬‬‬‬‬‬‬‬‬‬‬‬‬‬‬‬‬‬‬‬‬‬‬‬‬‬‬‬‬‬‬‬‬‬‬‬‬‬‬‬‬‬‬‬‬‬‬‬‬‬‬‬‬‬‬‬‬‬‬‬‬‬‬‬‬‬‬‬‬‬‬‬‬‬‬‬‬‬‬‬‬‬‬‬‬‬‬‬‬‬‬‬‬‬‬‬‬‬‬‬‬‬‬‬‬‬‬‬‬‬‬‬‬‬‬‬‬‬‬‬‬‬‬‬‬‬‬‬‬‬‬‬‬‬‬‬‬‬‬‬‬‬‬‬‬‬‬‬‬‬‬‬‬‬‬‬‬‬‬‬‬‬‬‬‬‬‬‬‬‬‬‬‬‬‬‬‬‬‬‬‬‬‬‬‬‬‬‬‬‬‬‬‬‬‬‬‬‬‬‬‬‬‬‬‬‬‬‬‬‬‬‬‬‬‬‬‬‬‬‬‬‬‬‬‬‬‬‬‬‬‬‬‬‬‬‬‬‬‬‬‬‬‬‬‬‬‬‬‬‬‬‬‬‬‬‬‬‬‬‬‬‬‬‬‬‬‬‬‬‬‬‬‬‬‬‬‬‬‬‬‬‬‬‬‬‬‬‬‬‬‬‬‬‬‬‬‬‬‬‬‬‬‬‬‬‬‬‬‬‬‬‬‬‬‬‬‬‬‬‬‬‬‬‬‬‬‬‬‬‬‬‬‬‬‬‬‬‬‬‬‬‬‬‬‬‬‬‬‬‬‬‬‬‬‬‬‬‬‬‬‬‬‬‬‬‬‬‬‬‬‬‬‬‬‬‬‬‬‬‬‬‬‬‬‬‬‬‬‬‬‬‬‬‬‬‬‬‬‬‬‬‬‬‬‬‬‬‬‬‬‬‬‬‬‬‬‬‬‬‬‬‬‬‬‬‬‬‬‬‬‬‬‬‬‬‬‬‬‬‬‬‬‬‬‬‬‬‬‬‬‬‬‬‬‬‬‬‬‬‬‬‬‬‬‬‬‬‬‬‬‬‬‬‬‬‬‬‬‬‬‬‬‬‬‬‬‬‬‬‬‬‬‬‬‬‬‬‬‬‬‬‬‬‬‬‬‬‬‬‬‬‬‬‬‬‬‬‬‬‬‬‬‬‬‬‬‬‬‬‬‬‬‬‬‬‬‬‬‬‬‬‬‬‬‬‬‬‬‬‬‬‬‬‬‬‬‬‬‬‬‬‬‬‬‬‬‬‬‬‬‬‬‬‬‬‬‬‬‬‬‬‬‬‬‬‬‬‬‬‬‬‬‬‬‬‬‬‬‬‬‬‬‬‬‬‬‬‬‬‬‬‬‬

### Statistical analysis

Kolmogorov–Smirnov tests were used to determine deviations from the normal distribution for all continuous variables, and 0.05 was regarded as a significant level for this test. After that, continuous variables with normal distribution were expressed as mean ± standard deviation (SD) and compared two groups using the student's *t*-test. Deviation from Hardy–Weinberg equilibrium (HWE) and allele frequency were checked using PLINK (version 1.9) [20]. Linkage disequilibrium (LD) heatmap was made by the LDheatmap package [22]in the R software.

By python programming, Fisher exact test analysis for comparing the allele frequency and Cox analysis for assessing the age of MI of participants carrying out the different alleles was done. Four different age classes, early (20–45 years), middle (45–65 years), late (65–80 years), and (80 < years), and two different sexes for all 17 SNPs were investigated by considering four genetics models (Additive, Dominant, Recessive, and Overdominant). The adjusted odds ratio (OR) was calculated, and the FDR adjusted *p *value (or *q *value) of 0.05 was applied [23]. The statistical analyses were performed with python programming and SPSS 24.0 (SPSS, Chicago, IL, USA).

## Results

**Fig. 1:**
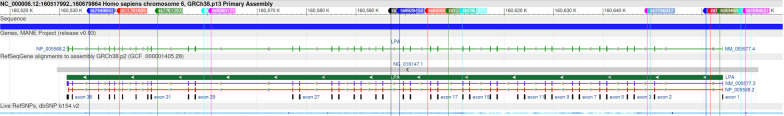
Genomic(intron/exome) structure of the LPA locus. Positions of the investigated Rs in this study are also marked on this locus

Among TCGS study participants, 783 individuals (459 Men and 323 Women; 21 to 92 years old) had experienced MI (men: 62.7 ± 11.1 years; women: 62.2  ± 10.5 years). The descriptive table of demographics and biochemical characteristics of the case and control groups is presented in Table 1. Table 2 describes different age classes. The genomic (intron/exome) structure of the *LPA* locus and location of the investigated Rs are also presented in Fig. 1. Moreover, the frequencies of the different alleles of the SNPs are shown in Table 3 for males and females.

**Table 1:** Baseline demographic and biochemical characteristics of the population

Unrelated individuals									
**Characteristics**	Male (*n* = 918)			Female (*n* = 646)			All (*n* = 1564)		
	Non-CHD (*n* =)	CHD (*n* =)	***P *value**^b^	Non-CHD (*n* =)	CHD (*n* =)	*P *value^b^	Non-CHD (*n* =)	CHD (*n* =)	***P *value**^b^
Age (years)	57 ± 11	57 ± 11	1	57 ± 9	57 ± 9	1	57 ± 10	57 ± 10	1
SBP (mm Hg)	122 ± 27	128 ± 30	0.001	123 ± 27	126 ± 30	0.26	122 ± 27	127 ± 30	0.001
DBP (mm Hg)	76 ± 15	79 ± 17	0.013	77 ± 15	79 ± 18	0.18	77 ± 15	79 ± 17	0.006
BMI (kg/m^2^)	25 ± 7	27 ± 6	0.002	27 ± 5	27 ± 6	0.86	26 ± 6	27 ± 6	0.01
Cholesterol (mg/dl)	220 ± 46	232 ± 48	< 0.001	218 ± 40	234 ± 49	< 0.001	219 ± 44	233 ± 49	< 0.001
Triglyceride (mg/dl)	193 ± 130	228 ± 140	0.001	168 ± 91	224 ± 147	< 0.001	183 ± 123	226 ± 156	< 0.001
LDL (mg/dl)	141 ± 35	150 ± 39	0.001	138 ± 34	151 ± 41	0.001	140 ± 35	150 ± 39	< 0.001
HDL (mg/dl)	42 ± 11	38 ± 9	< 0.001	43 ± 10	40 ± 11	< 0.001	42 ± 11	39 ± 10	< 0.001

^a^Characteristics based on Mean ± SE

^b^*P* value of t student or chi-square test between case and control groups, SBP, systolic blood pressure; DBP, diastolic blood pressure; BMI, body mass index; LDL, low-density lipoprotein cholesterol; HDL, high-density lipoprotein cholesterol. Indicates statistical significance (P < 0.05)

**Table 2:** Different age class range and case and control frequencies in each class

Age categories	Years old range	Male = 919		Female = 646	
		**Case**	**Control**	**Case**	**Control**
Early	20–45	29	29	15	15
Middle	45–65	231	231	180	180
Late	65–80	175	175	120	120
Old	> 80	25	24	8	8

**Table 3:** Investigated Rs in this study and case and control frequencies in male and female groups. All of these SNPs are intron_variant of the LPA gene

Variation	Position	Ref Allele	Alt Allele	Male = 919						Female = 646					
				**Control = 459**			**Case = 460**			**Control = 323**			**Case = 323**		
				***HomRef***	***Het***	***HomAlt***	***HomRef***	***Het***	***HomAlt***	***HomRef***	***Het***	***HomAlt***	***HomRef***	***Het***	***HomAlt***
rs7449650	6:160,536,082	T	G	52	199	208	59	186	214	50	128	145	33	140	150
rs11751605	6:160,542,198	T	C	437	22	0	439	19	1	318	5	0	307	16	0
rs7761293	6:160,549,931	G	A	148	242	69	162	211	87	124	147	52	104	166	53
rs6415084	6:160,559,298	T	C	115	186	158	84	238	138	78	154	91	75	162	86
rs9365171	6:160,560,704	C	A	156	204	99	142	230	88	121	141	61	116	156	50
rs7770628	6:160,597,142	C	T	119	190	150	98	219	143	69	153	101	77	168	77
rs6926458	6:160,598,834	A	G	237	189	33	220	196	44	160	126	37	167	134	21
rs6930542	6:160,604,515	T	C	453	3	0	448	3	0	315	2	0	319	0	0
rs13202636	6:160,608,696	T	C	234	189	33	219	196	44	160	126	37	167	134	21
rs7761377	6:160,611,449	A	G	162	175	99	152	169	96	103	124	66	115	137	47
rs10945682	6:160,648,909	G	A	166	195	95	158	210	92	109	148	65	115	161	47
rs7756317	6:160,649,497	C	T	459	0	0	459	1	0	321	2	0	322	1	0
rs1321196	6:160,660,810	C	T	94	205	160	92	215	153	66	145	112	47	163	113
rs1367211	6:160,661,663	T	C	71	194	194	70	203	187	45	138	140	39	144	139
rs9346833	6:160,663,610	C	T	102	218	138	109	219	131	76	152	95	64	159	99
rs783149	6:160,667,886	C	A	374	80	5	365	91	4	254	62	6	259	61	3
rs1084651	6:160,668,785	G	A	375	79	5	371	86	3	259	59	5	259	61	3

Table 4 shows the results of univariate analysis of the association between MI incidence with risk factors, including the allele frequencies of the variantrs6415084 in the OverDominant genetical model, sex, and age.

**Table 4:** Univariate analysis results of the association between MI incidence with risk factors including the allele frequencies of the variant rs6415084 in OverDominant genetical model, sex, and age

	Variables in the Equation					
	*B*	SE	Wald	*df*	Sig	Exp(*B*)
Sex	− 0.066	0.073	0.83	1	0.362	0.936
AgeClass_In_selexted_phase	− 1.242	0.072	297.095	1	0	0.289
rs6415084_OverDominant	0.197	0.072	7.582	1	0.006	1.218

FDR adjusted *p *value (or *q *value) of 0.05 level of statistical significance put aside 16 SNPs. The only rs6415084 in the overdominant genetic model showed significant association with the MI probability. The statistical results of this association are reported in Table 5.

**Table 5:** Fisher_exact statistical analysis of the association of the allele frequencies of the variant rs6415084 in OverDominant genetical model with a statistically significant impact on MI incidence

	AgeClass	HomoRef	Het	HomoAlt	*p*_value	Odds_Ratio (OR)
Female	Early	6	14	10	1.000	1
	Middle	90	177	93	1.000	1.02248
	Late	48	119	73	0.606	1.18149
	Old	9	5	2	0.282	7
Male	Early	12	25	21	1.000	0.86878
	Middle	107	209	146	0.000	2.13976
	Late	70	169	111	0.134	1.41073
	Old	10	21	17	0.244	0.42308

Cox analysis results revealed that the same marker also showed a statistically significant association with age-of-CHD-onset. Our results showed an association between MI incidence and the rs6415084 variant in the overdominant genetic model. In addition, as presented in Table 6, there is a strong association between the frequency of different alleles of rs6415084 with age-of-CHD-onset and MI incidence in middle-aged men. Thus, this association depends on the age and sex of cases.

**Table 6:** CoxPHFitter statistical analysis of the association of allele frequencies of the variant rs6415084 in OverDominant genetical model with the age of MI incidence

rs: 6,415,084 Model: OverDominant					CoxPHFitter									
Male	Age Class	Homo Ref	Het	Homo Alt	coef	exp(coef)	se(coef)	coef Lower 95%	coef Upper 95%	exp(coef) Lower 95%	exp(coef) Upper 95%	z	*p*	− log2(*p*)
Female	Early	6	14	10	0.24	1.28	0.52	− 0.78	1.26	0.46	3.54	0.47	0.64	0.64
	Middle	90	177	93	0.02	1.02	0.15	-0.27	0.31	0.76	1.36	0.12	0.91	0.14
	Late	48	119	73	0.08	1.08	0.18	− 0.28	0.43	0.75	1.54	0.42	0.68	0.56
	Old	9	5	2	1.08	2.93	0.71	− 0.32	2.48	0.72	11.89	1.51	0.13	2.92
Male	Early	12	25	21	0.03	1.03	0.38	− 0.71	0.77	0.49	2.15	0.07	0.94	0.09
	Middle	107	209	146	0.5	1.64	0.13	0.24	0.76	1.27	2.13	3.76	< 0.005	12.54
	Late	70	169	111	0.32	1.37	0.15	0.02	0.61	1.02	1.85	2.09	0.04	4.76
	Old	10	21	17	− 0.52	0.6	0.43	− 1.37	0.33	0.26	1.4	− 1.19	0.23	2.1

**Fig. 2:**
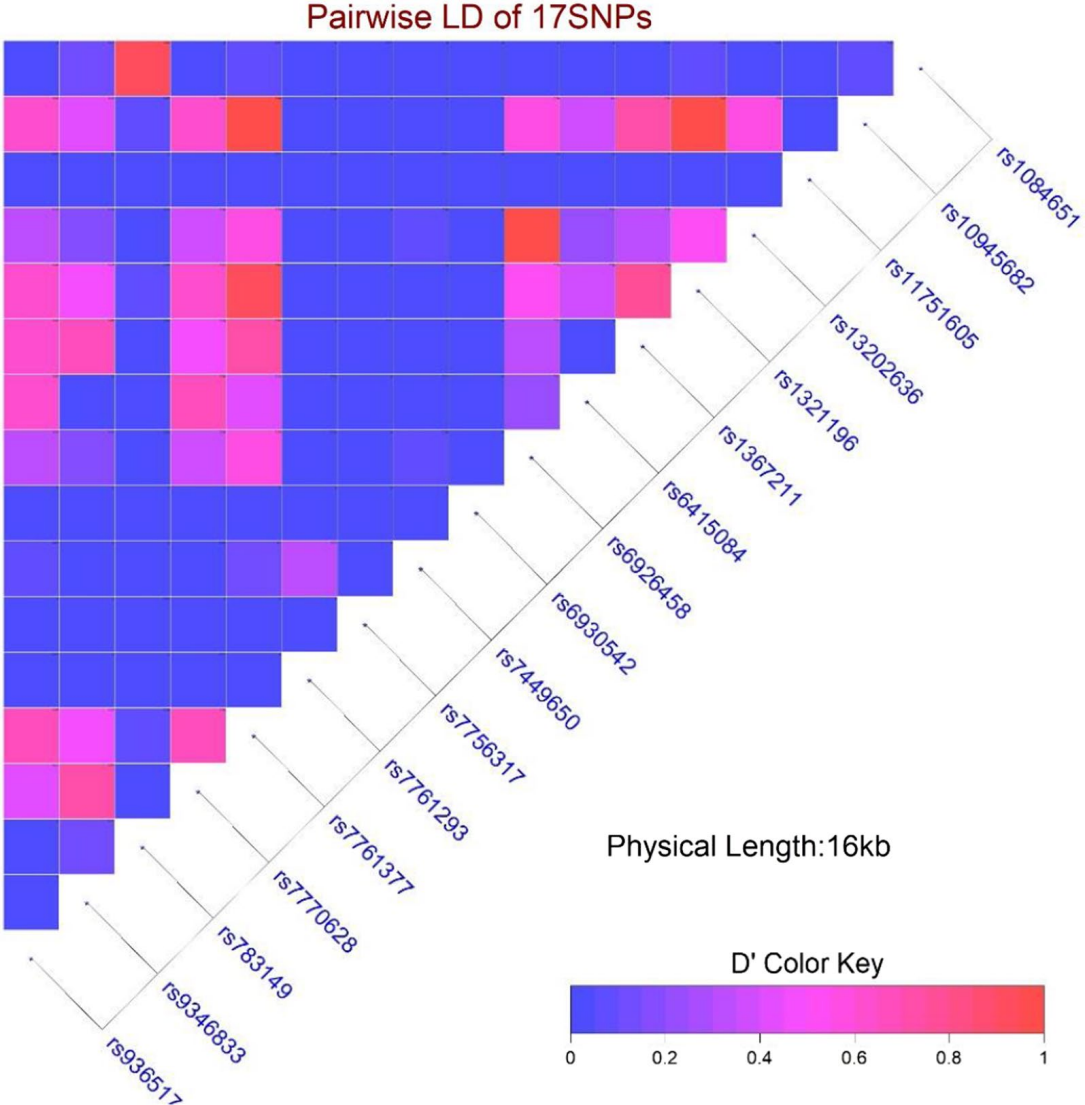
Linkage disequilibrium (LD) heatmap plot for all investigated SNPs in this study. This figure was produced by LDheatmap package in R software

rs6415084 did not show any significant linkage disequilibrium (LD) with other studied markers. Figure 2 and Additional file 1: Table S1.

To evaluate the associations between this genetic marker and other risk factors, univariate analysis results of the association between allele frequencies of the variant rs6415084 in OverDominant genetical model with risk factors including BMI, Cholesterol, TG, HDL, NHDL, and LDL. The parameters were obtained from the Pre-MI phase of each case.

## Discussion

The influence of age on Lp(a) concentrations is controversial. In the other studies, both older age and female sex are independent significant predictors of higher plasma Lp(a) [24, 25].

Base on our results, even the rs6415084 variants in the males that showed association with the largest age category did not show association with the early age-class variables. Thus, it is not only a sex-dependent but also an age-dependent association. The frequencies of 16 variants (rs7449650, rs11751605, rs7761293, rs9365171, rs7770628, rs6926458, rs6930542, rs13202636, rs7761377, rs10945682, rs7756317, rs1321196, rs1367211, rs9346833, rs783149, and rs1084651) did not associate with any of examined age–sex classes. These results confirmed the inter-population difference in genetics markers in agreement with our previous findings on the other genes variation on the TCGS data.

The importance of rs10455872 and its association with Lp(a) level reported on pharmacogenetics, CAD development in Brazilian patients, familial hypercholesterolemia studies, and Coronary artery calcification (CAC) [4, 6, 7, 9, 10]. In addition, the association of *LPA* polymorphism and carotid artery stenosis incidence, a significant association was observed for rs3798220. Contrastingly, no associations were detected for rs3798220 and rs10455872 and atherogenic stroke [8], carotid artery stenosis incidence with rs10455872, and CAD in Brazilian patients with rs3798220 [8, 9]. In addition, in a large-scale study, rs10455872 and rs3798220, have shown no associations with mortality in patients with prevalent coronary heart disease [11]. Moreover, a lack of associations between rs2048327, rs3127599, rs7767084, and rs10755578 with CAD in Chinese Han samples has also been reported [12, 13]. Besides, the Dumitrescu et al.'s study, which genotyped 19 *LPA* tag SNPs in 7,159 participants from the Third National Health and Nutrition Examination Survey (NHANES III), showed significant associations between Lp(a) concentration and *LPA* SNPs in non-Hispanic blacks than non-Hispanic whites and Mexican Americans. [14]. Interestingly, a lack of association between rs6415084 and subsequent cardiovascular events after the percutaneous coronary intervention has also been reported [13] in Chinese Han patients with coronary artery disease.

**Fig. 3:**
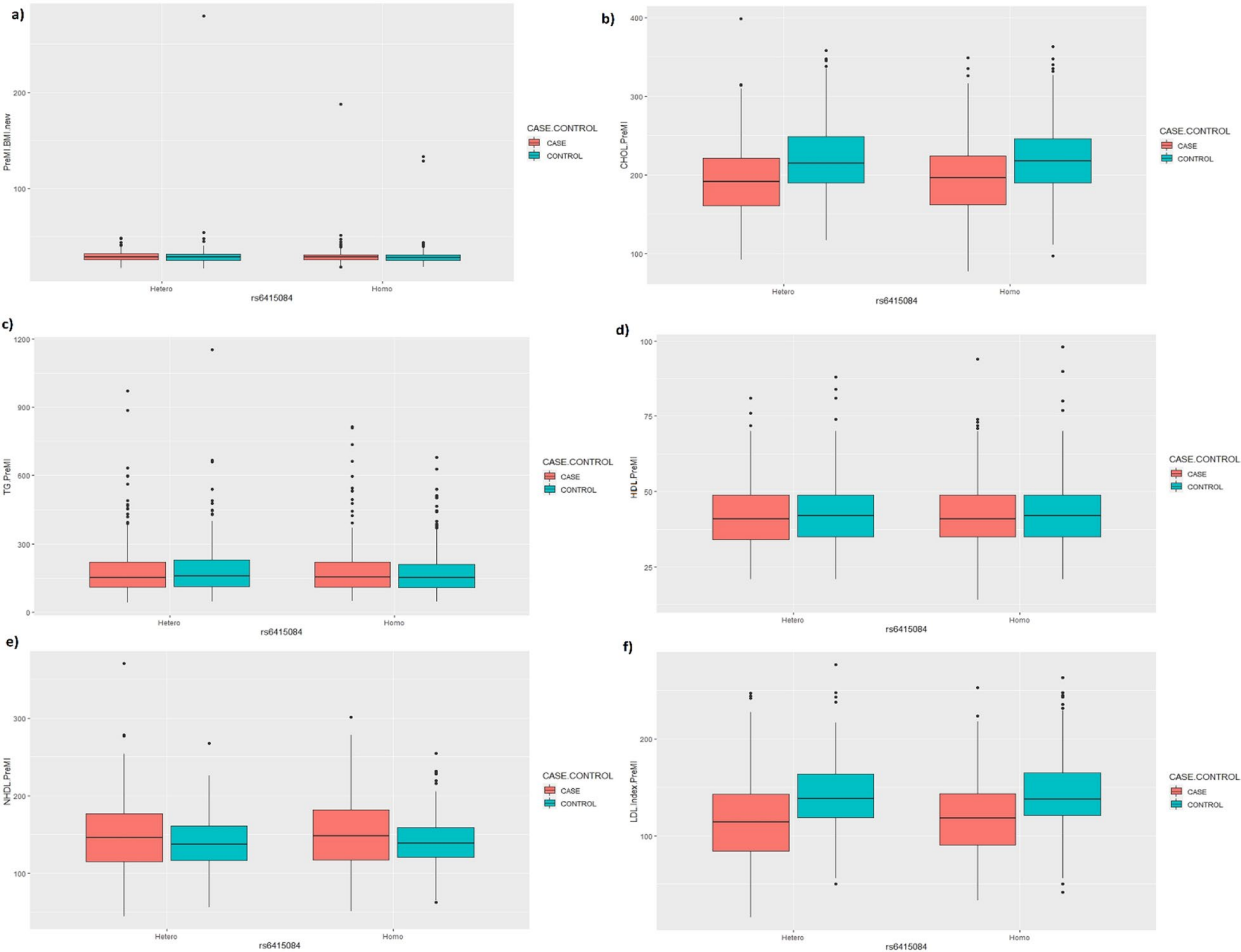
Univariate analysis results of the association between allele frequencies of the variant rs6415084 in OverDominant genetical model with risk factors, including **a** BMI, **b** Cholesterol, **c** TG, **d** HDL, **e** NHDL, and **f** LDL. There is a longitudinal study that every 3 years a new phase is started, so the above parameters were obtained from PreMI phase of each case

Genetic variation-based studies revealed the significant associations of variants in or near the *LPA* gene, with CHD risk incidence. Moreover, numerous case–control studies have confirmed that hyper-Lp(a) is a risk factor for premature cardiovascular disease [1, 2, 26]. Another study has shown that overweight and obesity are associated with significantly higher plasma Lp(a) [24]. Figure 3 shows the effect of the polymorphism on the plasma level of other risk factors such as obesity, LDL, HDL, etc. There was no statistically significant association between this polymorphism and these factors. This result proposes an independent relationship between the LP(a) factor and MI. This result is in agreement with the Paré et al. they also concluded that the *LPA* polymorphism association is independent of established MI risk factors, including diabetes mellitus, smoking, high blood pressure, and apolipoprotein B and A ratio. [27].

However, previous studies report the association of this polymorphism with the LP(a) level [28–30]; unfortunately, the Lp(a) level was not measured in this study.

These reports show a big controversy between the results of different studies and the importance of investigation of between-population differences in *LPA* genetic markers; in the same way, our results on the TCGS cohort also show specific association results; however, we found a sex and age dependency in the results.

### Perspectives and significance

This study emphasizes the population, age, and sex dependency of the associations, and none of the 17 SNPs showed significant differences between the case and control groups independent of age and sex.

## Conclusion

However, older patients are at an increased risk due to the human lifespan's natural limits. Still, our results show the genetic variation on the incidence of MI and the age-of-CHD-Onset. In addition, the population, age, and sex dependency of the association between the *LPA* variations and heart disease risk confirm that finding the effective variation in precision medicine is required to evaluate these factors in well-established cohorts. Thus, we propose the effects of these variants not only have differences among ethnic groups but also are sex and age-dependent.

## Supplementary Information

**Additional file 1: Table S1.** The results of r2 calculation of Linkagedisequilibrium (LD) for all investigated SNPs in this study.

## Data Availability

The data is available as additional files, and if the journal requires additional information, it is possible to send more details.
