# Supplementary material for: Sex, age, and ethnic dependency of lipoprotein variants as the risk factors of ischemic heart disease: a detailed study on the different age-classes and genders in Tehran Cardiometabolic Genetic Study (TCGS)
Source: Biol Sex Differ. 2022 Jan 28;13:4. doi: 10.1186/s13293-022-00413-7 (PMC8796330; doi:10.1186/s13293-022-00413-7)
Supplement: Supplementary file 1 — Additional file 1: Table S1. The results of r2 calculation of Linkagedisequilibrium (LD) for all investigated SNPs in this study. [file 13293_2022_413_MOESM1_ESM.docx]

**Title:** Age and sex dependency of myocardial infarction markers: A detailed study on the different age-classes and genders in Tehran Cardiometabolic Genetic Study (TCGS)

|  | rs9365171 | rs9346833 | rs783149 | rs7770628 | rs7761377 | rs7761293 | rs7756317 | rs7449650 | rs6930542 | rs6926458 | rs6415084 | rs1367211 | rs1321196 | rs13202636 | rs11751605 | rs10945682 | rs1084651 |
| --- | --- | --- | --- | --- | --- | --- | --- | --- | --- | --- | --- | --- | --- | --- | --- | --- | --- |
| rs9365171 | 0.000 | 0.000 | 0.000 | 0.406 | 0.673 | 0.007 | 0.000 | 0.069 | 0.002 | 0.328 | 0.635 | 0.634 | 0.617 | 0.328 | 0.009 | 0.629 | 0.000 |
| rs9346833 | 0.000 | 0.000 | 0.131 | 0.718 | 0.452 | 0.000 | 0.003 | 0.000 | 0.002 | 0.180 | 0.000 | 0.668 | 0.467 | 0.180 | 0.000 | 0.446 | 0.122 |
| rs783149 | 0.000 | 0.131 | 0.000 | 0.000 | 0.057 | 0.000 | 0.010 | 0.000 | 0.000 | 0.042 | 0.000 | 0.041 | 0.066 | 0.042 | 0.000 | 0.066 | 0.925 |
| rs7770628 | 0.406 | 0.718 | 0.000 | 0.000 | 0.679 | 0.012 | 0.002 | 0.024 | 0.002 | 0.364 | 0.656 | 0.486 | 0.622 | 0.364 | 0.003 | 0.646 | 0.000 |
| rs7761377 | 0.673 | 0.452 | 0.057 | 0.679 | 0.000 | 0.001 | 0.004 | 0.104 | 0.004 | 0.552 | 0.429 | 0.743 | 0.929 | 0.552 | 0.007 | 0.956 | 0.066 |
| rs7761293 | 0.007 | 0.000 | 0.000 | 0.012 | 0.001 | 0.000 | 0.002 | 0.313 | 0.004 | 0.045 | 0.030 | 0.000 | 0.000 | 0.045 | 0.025 | 0.001 | 0.000 |
| rs7756317 | 0.000 | 0.003 | 0.010 | 0.002 | 0.004 | 0.002 | 0.000 | 0.000 | 0.002 | 0.000 | 0.001 | 0.005 | 0.004 | 0.000 | 0.000 | 0.004 | 0.011 |
| rs7449650 | 0.069 | 0.000 | 0.000 | 0.024 | 0.104 | 0.313 | 0.000 | 0.000 | 0.000 | 0.060 | 0.010 | 0.000 | 0.000 | 0.060 | 0.009 | 0.000 | 0.000 |
| rs6930542 | 0.002 | 0.002 | 0.000 | 0.002 | 0.004 | 0.004 | 0.002 | 0.000 | 0.000 | 0.001 | 0.003 | 0.005 | 0.004 | 0.001 | 0.000 | 0.004 | 0.000 |
| rs6926458 | 0.328 | 0.180 | 0.042 | 0.364 | 0.552 | 0.045 | 0.000 | 0.060 | 0.001 | 0.000 | 0.208 | 0.316 | 0.548 | 0.999 | 0.004 | 0.564 | 0.000 |
| rs6415084 | 0.635 | 0.000 | 0.000 | 0.656 | 0.429 | 0.030 | 0.001 | 0.010 | 0.003 | 0.208 | 0.000 | 0.000 | 0.384 | 0.208 | 0.005 | 0.395 | 0.000 |
| rs1367211 | 0.634 | 0.668 | 0.041 | 0.486 | 0.743 | 0.000 | 0.005 | 0.000 | 0.005 | 0.316 | 0.000 | 0.000 | 0.764 | 0.316 | 0.000 | 0.738 | 0.049 |
| rs1321196 | 0.617 | 0.467 | 0.066 | 0.622 | 0.929 | 0.000 | 0.004 | 0.000 | 0.004 | 0.548 | 0.384 | 0.764 | 0.000 | 0.548 | 0.000 | 0.970 | 0.062 |
| rs13202636 | 0.328 | 0.180 | 0.042 | 0.364 | 0.552 | 0.045 | 0.000 | 0.060 | 0.001 | 0.999 | 0.208 | 0.316 | 0.548 | 0.000 | 0.004 | 0.565 | 0.039 |
| rs11751605 | 0.009 | 0.000 | 0.000 | 0.003 | 0.007 | 0.025 | 0.000 | 0.009 | 0.000 | 0.004 | 0.005 | 0.000 | 0.000 | 0.004 | 0.000 | 0.007 | 0.000 |
| rs10945682 | 0.629 | 0.446 | 0.066 | 0.646 | 0.956 | 0.001 | 0.004 | 0.000 | 0.004 | 0.564 | 0.395 | 0.738 | 0.970 | 0.565 | 0.007 | 0.000 | 0.062 |
| rs1084651 | 0.000 | 0.122 | 0.925 | 0.000 | 0.066 | 0.000 | 0.011 | 0.000 | 0.000 | 0.000 | 0.000 | 0.049 | 0.062 | 0.039 | 0.000 | 0.062 | 0.000 |

**Table S1: The results of r^2 calculation of Linkage disequilibrium (LD) for all investigated SNPs in this study.**
